# Solution Process-Based Thickness Engineering of InZnO Semiconductors for Oxide Thin-Film Transistors with High Performance and Stability

**DOI:** 10.3390/mi15020193

**Published:** 2024-01-27

**Authors:** Xuan Zhang, Sung-Woon Cho

**Affiliations:** Department of Advanced Components and Materials Engineering, Sunchon National University, Sunchon 57922, Republic of Korea; 1215065@s.scnu.ac.kr

**Keywords:** oxide semiconductor, thin-film transistor, thickness, solution process, high performance, high stability

## Abstract

To fabricate oxide thin-film transistors (TFTs) with high performance and excellent stability, preparing high-quality semiconductor films in the channel bulk region and minimizing the defect states in the gate dielectric/channel interfaces and back-channel regions is necessary. However, even if an oxide transistor is composed of the same semiconductor film, gate dielectric/channel interface, and back channel, its electrical performance and operational stability are significantly affected by the thickness of the oxide semiconductor. In this study, solution process-based nanometer-scale thickness engineering of InZnO semiconductors was easily performed via repeated solution coating and annealing. The thickness-controlled InZnO films were then applied as channel regions, which were fabricated with almost identical film quality, gate dielectric/channel interface, and back-channel conditions. However, excellent operational stability and electrical performance suitable for oxide TFT backplane was only achieved using an 8 nm thick InZnO film. In contrast, the ultrathin and thicker films exhibited electrical performances that were either very resistive (high positive *V_Th_* and low on-current) or excessively conductive (high negative *V_Th_* and high off-current). This investigation confirmed that the quality of semiconductor materials, solution process design, and structural parameters, including the dimensions of the channel layer, must be carefully designed to realize high-performance and high-stability oxide TFTs.

## 1. Introduction

Multicomponent amorphous oxide semiconductors (for example, InGaZnO, InZnO, and ZnSnO), including indium oxide, zinc oxide, tin oxide, and their composites, are promising channel materials for thin-film transistors (TFTs) that perform driving and switching functions in display backplanes [1,2]. Compared to the existing amorphous Si, these semiconductors have various material properties, such as excellent electrical conduction path, wide band gap, and large-area deposition uniformity, which are suitable for the manufacture of high-performance TFTs for next-generation high-resolution and large-size displays [3,4,5]. Therefore, to implement high-performance oxide transistors based on the aforementioned advantages, the following main factors related to the channel bulk region must be optimized: (i) the oxide semiconductor thin film in the channel bulk region, and (ii) the interface and surfaces surrounding the channel bulk region. For a high-quality channel bulk region, various film deposition processes (vacuum- and solution-based) [6,7,8,9] and post-treatment techniques (thermal annealing, plasma treatment, microwave treatment, and electron beam treatment) [10,11,12,13] have steadily been developed to fabricate high-quality oxide semiconductor films with an excellent M–O–M network, appropriate oxygen vacancies, and low impurities.

However, even though high-quality oxide semiconductors are prepared in the channel region, the device performance is significantly influenced by the contribution of the gate dielectric/channel interfaces and back-channel regions [14,15]. Generally, semiconducting oxide thin films with thicknesses ranging from a few nanometers to several tens of nanometers are used in the channel region of oxide TFTs. Thus, the carriers present in the channel region frequently interact with the gate dielectric/channel interface and the channel surface (back channel) during charge transporting. In particular, the area surrounding the bulk channel region includes many charge-trapping sites that cause electrical degradation and device instability. Therefore, several methods based on physical and chemical approaches and structural design have been proposed to suppress these problems. For physical and chemical approaches, the development of interface-treatment technologies and surface-passivation layers has been continuously conducted to suppress the active defect sites present at the gate dielectric/channel interface and back-channel region [16,17,18]. Next, by appropriately designing the channel structure (such as channel size, channel thickness, and stacking structure), the degree of interaction between the carriers and defect states at the interfaces and surfaces can be engineered [19,20,21]. The channel size can significantly influence TFT performance. In general, when the channel length is short, TFTs show lower mobility and more conductive electrical properties [19,20]. In addition, the channel thickness is an important factor. For thinner semiconductor films, free carriers frequently interact with the interfaces and surfaces, implying that the TFT will exhibit lower device performance and stability. However, in the case of an excessively thick semiconductor film, controlling the number of free electrons in the channel region by applying a bias to the gate terminal is challenging. The electrical performance and stability of oxide TFTs can vary with nanometer-scale changes in the channel layer thickness. Therefore, forming an oxide semiconductor with an optimal thickness in the channel region is crucial.

Generally, oxide semiconductor films are manufactured using a vacuum-based deposition process. Compared to solution-based processes, the film thickness can be easily controlled on the nanometer scale when using vacuum-based deposition processes [22,23]. Furthermore, the film thickness could be properly controlled by changing the growth time. Therefore, several studies could explore the change in the performance according to the channel thickness of devices based on vacuum-processed oxide semiconductors [24,25]. However, in solution-processed cases, investigations exploring nanometer-scale thickness engineering of oxide semiconductors for oxide TFTs with high performance and stability are limited. In particular, it is difficult to control the thickness of an oxide semiconductor on a nanometer scale through printing processes such as inkjet, gravure, and roll-to-roll, due to the coffee ring effect [26,27]. In contrast, the spin coating method enables nanometer-scale thickness control of oxide semiconductors by adjusting the concentration of the precursor solution, spin-coating speed, and repeated coating and annealing. Here, the thickness control according to the spin coating speed is generally insignificant. Next, controlling the concentration of the precursor solution affects both the film thickness and density [28]. However, repeated coating and annealing is the most suitable method for controlling the film thickness at the nanoscale while maintaining the quality of the semiconductor film [13,29,30].

In this study, we performed nanometer-scale thickness engineering of oxide semiconductor films via spin-coating of precursor solutions. Nanometer-scale thickness engineering of oxide semiconductor films can be achieved via repeated coating and annealing. InZnO thin films with thicknesses ranging from 4 to 16 nm can be fabricated in the channel region. Regardless of the thickness, the films applied to the channel bulk region were confirmed to have similar film quality based on the chemical bonding states of the metal cations and oxygen anions. Microstructural surface analysis revealed that the back-channel region had a similar quality. However, even if the oxide transistor is composed of a bulk channel (i.e., InZnO thin film), gate dielectric/channel interface, and back channel of similar quality, the electrical performance and operational stability are significantly affected by the channel thickness.

## 2. Experimental Details [31]

### 2.1. Film and Device Fabrication

Precursor solutions for InZnO films were prepared by dissolving zinc nitrate hydrate (Zn(NO_3_)_2_·*x*H_2_O; Sigma-Aldrich, St. Louis, MO, USA) and indium nitrate hydrate (In(NO_3_)_3_·*x*H_2_O; Sigma-Aldrich) in 2-methoxyethanol (CH_3_OCH_2_CH_2_OH; Sigma-Aldrich). Next, 0.1 M InZnO precursor solution was produced with equivalent compositional ratios between In and Zn (In:Zn = 5:5). The synthesized precursor solutions were mixed for 1 h at room temperature using a sonicator until they were completely ionized. Before spin coating, the solutions were filtered through a hydrophobic 0.2-μm syringe filter. An individual InZnO precursor solution was deposited by spin coating at 3000 rpm for 30 s onto heavily doped *p*^++^-Si wafers with 200 nm thick SiO_2_ layers; the heavily doped *p*^++^-Si wafers and SiO_2_ layers served as the gate electrode and gate dielectric, respectively. The semiconducting InZnO film with superior metal oxide-bonding networks was produced via sub-sequential soft- (200 °C for 10 min) and hard-bake processes (400 °C for 1 h) using hot-plates. The spin-coating and baking processes were repeated one to four times to control the thickness of the InZnO thin film. Next, as source and drain electrodes for TFTs, 100 nm thick Al films were deposited by thermal evaporation and defined via conventional liftoff processing. The channel dimensions were 500 µm in width and 50 µm in length.

### 2.2. Film and Device Characteristics

The chemical bonding states of the InZnO films were evaluated through X-ray photoelectron spectroscopy (XPS; ESCALAB 250, Thermo Fisher Scientific, Waltham, MA, USA) using an Al Kα (1486.6 eV) source. The thickness and surface morphology of the InZnO films were measured using non-contact-mode atomic force microscopy (AFM; NX-10, Park Systems, Suwon, Republic of Korea).

The electrical performances of the solution-processed InZnO TFTs were evaluated using a semiconductor parameter analyzer (HP-4145B, Agilent Technologies, Santa Clara, CA, USA) under dark conditions at room temperature in ambient air. The *I_DS_*–*V_GS_* transfer curve was measured with *V_GS_* from −40 to +40 V at specific *V_DS_* = 10 V. The field effect mobility (*μ_FE_*) was extracted from the transfer curve in the linear regime (*V_GS_* − *V_Th_* ≫ *V_DS_*, *V_DS_* = 1 V) using the following equation [24]:

(1) $$\mu_{FE}={\left[\frac{L}{C_{i}\;W{\;V}_{DS}}\frac{{dI}_{DS}}{{dV}_{GS}}\right]}_{max},$$

where *I_DS_*, *V_DS_*, *V_GS_*, *C_i_*, *W*, and *L* represent the drain current, applied drain voltage, applied gate voltage, gate dielectric capacitance per unit area, channel width, and channel length, respectively.

The threshold voltage (*V_Th_*) was estimated from the transfer curve in the saturation regime (*V_DS_* = 10 V) via the following equation [32]:

(2) $$I_{DS}=\left(\frac{W}{2L}\right)\mu_{SAT}\;C_{i}\;{\left(V_{GS}-V_{Th}\right)}^{2},$$

where *μ_SAT_* is the saturation mobility. The *V_Th_* was determined from the *x*-axis intercept of the √(*I_DS_*) versus *V_GS_* plot by linear extrapolation.

The sub-threshold swing value (*S.S.*) was estimated from the transfer curve in the saturation regime (*V_DS_* = 10 V) via the following equation [24]:

(3) $$S.S.={\left[{\left(\frac{{dI}_{DS}}{{dV}_{GS}}\right)}_{max}\right]}^{-1}.$$

The density of the charge-trapping states (*N_T_*) in the gate dielectric/channel interface was estimated from the hysteresis curve via the following equation [33]:

(4) $$N_{T}=C_{i}\;\frac{∆V_{Hys}}{q}.$$

where *C_i_*, ∆*V_Hys_*, and *q* are the gate dielectric capacitance per unit area, difference of *V_Th_* extracted on forward and backward transfer curves, and elementary charge, respectively.

## 3. Results and Discussion

**Figure 1:**
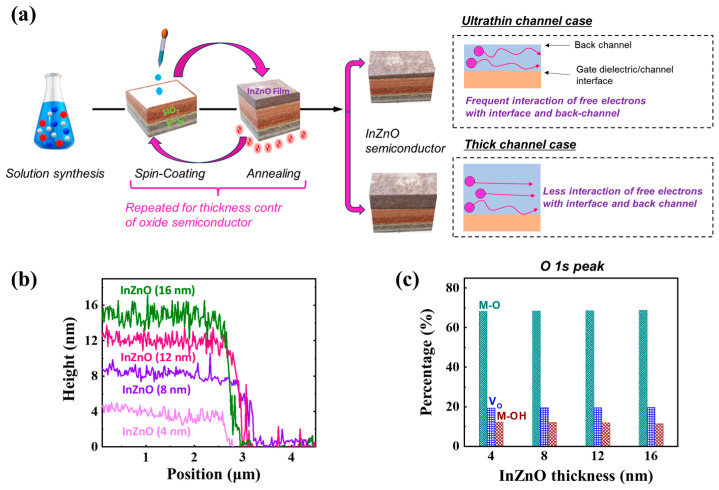
(**a**) Fabrication scheme of solution-processed InZnO semiconductor films and thickness control via repeated coating and annealing, (**b**) film thickness date (measured by AFM), and (**c**) chemical bonding states (metal-oxygen (M-O), oxygen vacancy (VO), and metal hydroxide (M-OH)) of thickness-controlled InZnO films.

Figure 1a shows the coating-based thickness engineering procedure of InZnO semiconductors to fabricate InZnO oxide TFTs with high performance and excellent operational stability. The InZnO semiconductors with various film thickness could be fabricated by repeatedly coating and annealing the precursor solution. InZnO films with thicknesses of 4, 8, 12, and 16 nm were prepared by repeating the processing one, two, three, and four times, respectively. As shown in Figure 1b and Figure S1, the film thickness was evaluated by measuring the edge height of the patterned InZnO films. The thermal energy for the chemical reaction from the precursor solution to the oxide film was supplied at the same temperature (400 °C), regardless of the thickness of the InZnO thin film. Thus, as shown in Figure 1c and Figure S2, all the films were composed of excellent M–O–M networks with high ratios of M–O bonds. Regardless of the thickness, the films had similar distributions of M–O bonds and oxygen vacancies. The M–O–M networks of the InZnO films can be estimated by deconvolution of the O 1s peaks measured via XPS analysis. In addition, regardless of the film thickness, the surface morphology of the InZnO film with amorphous phase was similar, which was confirmed by the surface roughness values (Figure S1); the surface roughness of InZnO films with thicknesses 4, 8, 12, and 16 nm was 0.13, 0.12, 0.16, and 0.17 nm, respectively. In addition, an InZnO film was spin-coated and post-annealed on the gate dielectric under the same conditions, indicating that the quality of the gate dielectric/semiconductor interface was similar. Nevertheless, as shown in Figure 1a, the electrical characteristics of InZnO TFTs can be engineered significantly depending on the film thickness of the InZnO semiconductors employed in the channel region. In ultrathin InZnO films, electrical carriers frequently experience charge trapping at interfaces and back channels during device operation, contributing to performance degradation and operational instability. In contrast, for thicker semiconductor films, electrical carriers are effectively transferred with minimal interaction with the gate dielectric/channel interface and the back channel. This is advantageous for achieving the high performance and stability of the TFT device.

**Figure 2:**
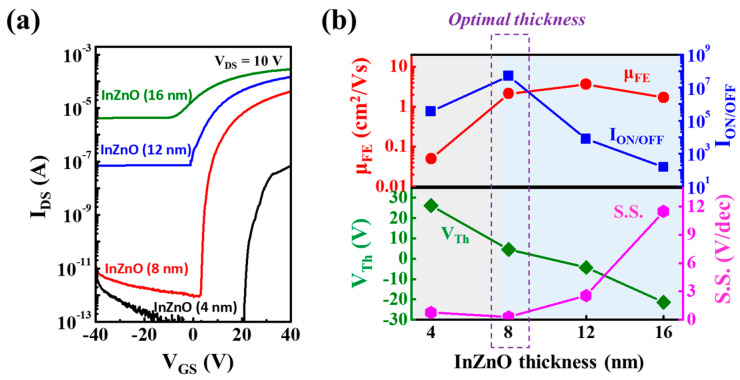
(**a**,**b**) Electrical characteristics of TFTs fabricated using thickness-controlled InZnO semiconductors: (**a**) *I_DS_*–*V_GS_* transfer curve and (**b**) figure-of-merits (*I_ON/Off_*, *μ_FE_*, *V_Th_*, and *S.S.*).

As shown in Figure 2, various InZnO TFTs were fabricated by introducing InZnO semiconductor layers with different thicknesses (4, 8, 12, and 16 nm) in the channel region. Here, 11 devices were fabricated for each channel thickness condition (Figure S3). The electrical performances such as *μ_FE_*, *V_Th_*, on/off current ratio, and S.S. were then extracted from the transfer curves of the 11 devices (Figures S4–S6). As shown in Figure 2a, all the InZnO TFTs exhibited the operational behavior of TFTs using n-type semiconductors (off state on negative gate bias and on state on positive gate bias). For ultrathin InZnO films (thickness of 4 nm), oxide TFT with poor charge transport and on/off switching capability was produced, despite having conduction paths of M–O–M network quality similar to those in other films. In the case of an ultrathin InZnO film with a thickness of 4 nm, free electrons in the narrow-channel region are frequently trapped at the gate dielectric/channel interface and back channel. In contrast, as shown in Figure 2b, excellent electrical performance, such as high on/off current ratio (*I_ON/OFF_* = ~10^7^), superior mobility (*μ_FE_* = 2.1 cm^2^/V·s), and low sub-threshold swing value (*S.S.* = 0.289), was achieved using the thin InZnO film with thickness of 8 nm. As shown in Figure 2a, under conditions of zero or negative gate bias, no channel layer is formed in the thin InZnO film with a thickness of 8 nm due to the low initial free-electron concentration, which is similar to that when using ultrathin InZnO with a thickness of 4 nm. Thus, a low-off-current state can be easily realized under low negative gate bias conditions. Next, the channel region can be formed under lower positive gate bias conditions compared to when using ultrathin InZnO. This is because more free electrons can accumulate in the thicker InZnO layers when an identical gate bias is applied. Thus, the threshold voltage when using 8 nm thick InZnO (*V_Th_* = 0.8 V) was lower than that of when using ultrathin InZnO with a thickness of 4 nm (*V_Th_* = 22 V). In addition, the free electrons present in a wider channel region encounter charge-trapping sites with a relatively low probability. Thus, individual carriers can be efficiently transported with less influence from charge-trapping sites located at the interfaces and surface regions, compared to when using ultrathin InZnO. Similarly, as shown in Figure 2a, when thickness is greater than 8 nm, free electrons can be effectively transported further following the percolation conduction mechanism, with less influence from interfacial and surface traps and a higher accumulated carrier concentration. However, the excessive initial free electrons present in the bulky InZnO films, compared to that in the optimal case, resulted in an overall negative shift in the transfer curve and a high off-current state. The observed performance degradation is likely attributed to the elevated concentration of free electrons in the bulky InZnO films. Additionally, the subthreshold swing (*S.S.*) also deteriorates, especially in the case of the 16 nm thick InZnO film. This is due to the introduction of numerous defects through multiple spin-coating steps, leading to the degradation of interface states. By conducting comparative experiments using devices fabricated with a single spin-coating of precursor solution with concentrations of 0.1 M (4 nm), 0.2 M (8 nm), 0.3 M (12 nm), and 0.4 M (16 nm), as illustrated in Figure S7, it is evident that the performance of devices with the same thickness of InZnO film is lower when using a single spin-coating compared to multiple spin-coatings. Despite the instability and significant reduction in performance of devices with a single spin-coating, they still exhibit a noticeable increase in off-current state with the rise in concentration of the IZO precursor solution. This suggests that the overall negative shift in the transmission curve and the high off-current state caused by increased thickness are attributed to the elevated concentration of free electrons in the bulky InZnO film. These factors collectively have adverse effects on the characteristics of excessively thick film transistors, such as an increase in off-state current and a decrease in subthreshold swing. Applying an oxide TFT with a depletion-mode operation and high off-current level is disadvantageous for power savings and good on/off switching operations. Hence, among the thickness-controlled InZnO films, an InZnO thin film with a thickness of 8 nm was the most appropriate for fabricating high-performance oxide TFT.

During display operation, the switching and driving TFTs on the TFT backplane for liquid-crystal and organic light-emitting diode displays are constantly exposed to several stress factors. They experience repetitive positive and negative gate bias stresses for on/off switching and output amplification, thermal stress due to self-heating, and/or light stress caused by the surrounding light sources (organic light-emitting diodes and backlight units). Oxide TFTs exhibit unstable electrical characteristics under these stress factors, which appear as negative and positive shifts in the transfer curve. Generally, oxide TFTs have transfer curves that move in the negative direction under thermal and illumination stresses. The transfer curve tended to move in the positive and negative directions under positive bias stress (PBS) and negative bias stress (NBS) [34,35,36], respectively. As shown in Figure S8, a NBS test using a gate bias stress condition of −10 V for 7.2 ks was performed to evaluate the operational stability of oxide TFTs using thickness-controlled InZnO thin films. In Figure S8a, the results are presented, indicating that the TFT with an ultrathin InZnO film of 4 nm thickness exhibits a significant positive shift in the transmission curve. On the other hand, TFTs with thicker InZnO layers show a slight negative shift. The observed positive shift in the transmission curve for the ultrathin film can be attributed to its low free electron concentration. In the wider channel region of the ultrathin film, there is a relatively high probability of free electrons encountering charge trapping sites, leading to the observed positive shift in the TFT response. This behavior suggests that the operational stability of TFTs is influenced by the thickness of the InZnO thin films. In particular, the transfer curve was more sensitive to PBS than to NBS. This is because the free electrons are actively captured at the charge-trapping sites under PBS conditions: (i) defect states present at the gate dielectric/channel interface, and (ii) electron/oxygen gas molecular interactions in the back-channel region [15,37].

**Figure 3:**
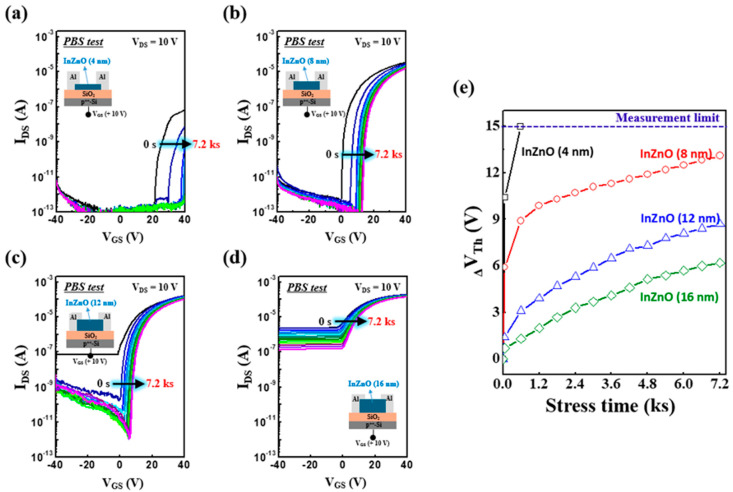
(**a**–**e**) Transfer curve shifts of TFTs fabricated using thickness-controlled InZnO semiconductors under PBS tests: (**a**) 4 nm thick InZnO, (**b**) 8 nm thick InZnO, (**c**) 12 nm thick InZnO, (**d**) 16 nm thick InZnO, and (**e**) the summarized result.

**Figure 4:**
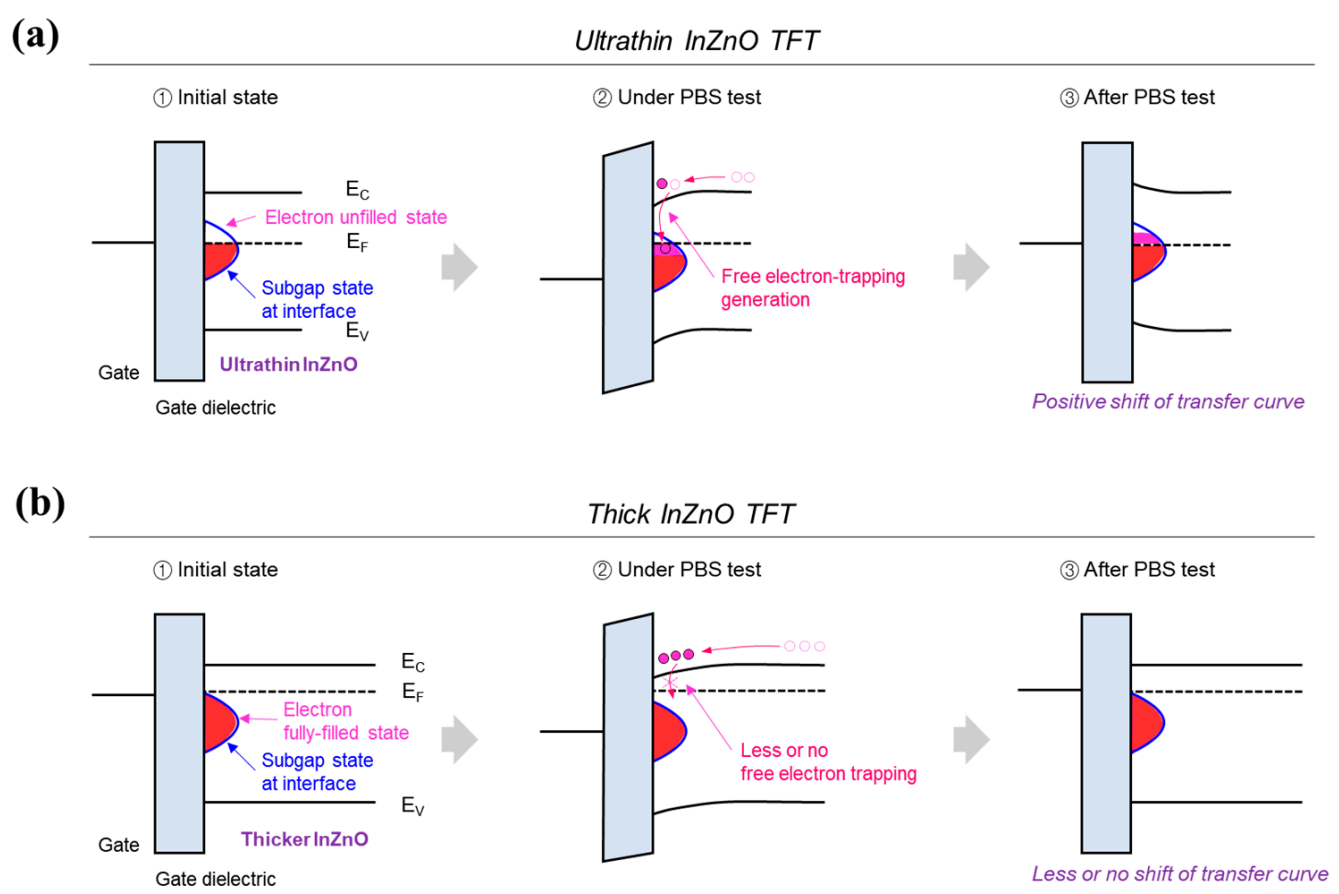
(**a**,**b**) Charge-trapping behaviors exhibited near the gate dielectric/channel interface of TFTs using (**a**) ultrathin and (**b**) thick InZnO films under the PBS test.

As shown in Figure 3a, a PBS test using a gate bias stress condition of +10 V for 7.2 ks was performed to evaluate the operational stability of oxide TFTs using thickness-controlled InZnO thin films. All the TFTs, regardless of the thickness of the InZnO film, have similar gate dielectric/channel interfaces and back-channel conditions, indicating that they contain almost the same charge-trapping sites. However, as shown in Figure 3a, the TFT with a 4 nm thick InZnO film exhibited a significant positive shift in the transfer curve despite a short stress time. The transfer curve shifts excessively in the positive direction beyond the measurement range during the PBS test. This is because free electrons in the narrow-channel region are frequently trapped at the active subgap states present in the gate dielectric/channel interface or can easily interact with the surrounding oxygen gas molecules at the back channel. As shown in Figure 4a, when the charge-trapping defect states near the gate dielectric/channel interface were not filled with electrons, they actively captured free electrons under the PBS stress condition, causing a dramatic positive directional shift in the transfer curve. In contrast, as shown in Figure 3b–e, the instability under PBS stress can be overcome by increasing the thickness of the InZnO film. The ∆*V_Th_* of TFTs under PBS is 13.1, 8.7, and 6.2 V for InZnO films with a thickness of 8, 12, and 16 nm. As shown in Figure 4b, the thicker semiconducting films contained more electron carriers. Thus, in the initial state, many subgap states that contribute to charge trapping at the gate dielectric/channel interface are already filled. These electron-filled subgap states do not serve as active electron-trapping sites in the PBS test. This indicates that fewer active trapping sites exist in thicker InZnO films, despite having almost equal amounts of charge-trapping sites near the gate dielectric/channel interface and the back channel. Thus, the shift in the positive direction of the transfer curve in the PBS test was significantly reduced when the thick InZnO films were used. However, the deterioration of the interface state in thicker InZnO films is evident due to the excessive application of multiple spin-coating steps. Therefore, the utilization of thicker InZnO films leads to a notable reduction in the positive shift of the transmission curve during PBS testing. Conversely, the sub-threshold swing (*S.S.*) experiences severe degradation under these conditions. Hence, whether the film thickness is ultrathin or ultrathick, the device performance tends to decrease. As shown in Figure 2b, that optimal performance is achieved at a thickness of 8 nm.

**Figure 5:**
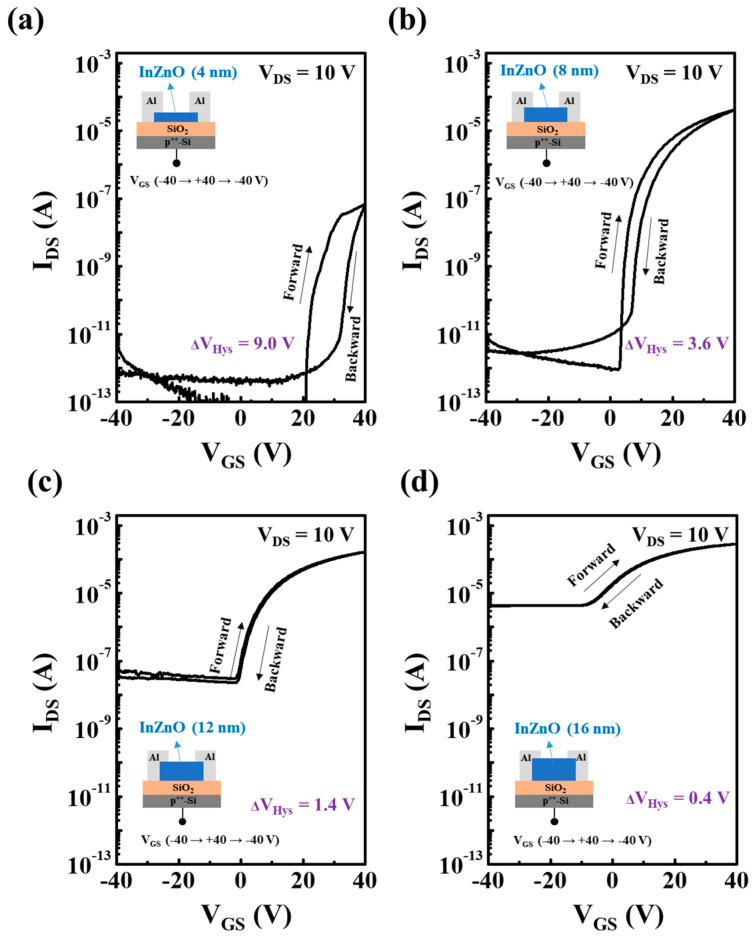
Hysteresis properties of InZnO TFTs using thickness-controlled InZnO semiconductors with film thicknesses of (**a**) 4, (**b**) 8, (**c**) 12, and (**d**) 16 nm.

Figure 5 shows the hysteresis curves of the oxide TFTs with thickness-controlled InZnO films. The hysteresis value decreased as the thickness of the InZnO thin film increased; ∆V_Hys_ in TFTs with InZnO films with thicknesses of 4, 8, 12, and 16 nm was 9.0, 3.6, 1.4, and 0.4 V, respectively. This indicates that the TFT with ultrathin InZnO contains many active trapping sites at the gate dielectric/channel interface. Meanwhile, few active trapping sites were present in the gate dielectric/channel interface region of the TFTs with thick InZnO films. The densities of the active trapping states present near the gate dielectric/channel interface estimated from the hysteresis curves were 9.71 × 10^11^, 3.88 × 10^11^, 1.51 × 10^11^, and 4.32 × 10^10^ cm^−2^ for TFTs using 4, 8, 12, and 16 nm thick InZnO films, respectively. Therefore, the active trapping state of the gate dielectric/channel region contributes significantly to the instability of the oxide TFTs in the PBS test. Finally, among the thickness-controlled InZnO thin films, the 8 nm thick InZnO semiconductor thin film was confirmed to be the most suitable for fabricating oxide TFTs with excellent performance and operational stability.

## 4. Conclusions

In order to fabricate high-performance oxide TFTs with excellent stability, preparing high-quality semiconductor films with good M–O–M networks, appropriate oxygen vacancies, and low defect states is necessary. Additionally, the defect states present at the gate dielectric/channel interfaces and back channels must be suppressed. However, even if the oxide transistor is composed of the same quality semiconductor film, gate dielectric/channel interface, and back channel, the electrical performance and operational stability of the oxide TFT are greatly affected by the thickness of the oxide semiconductor. The film thickness of the InZnO semiconductors can be easily engineered via repeated solution coating and annealing. The thickness-controlled InZnO films were then applied as channel regions for oxide TFTs, which were fabricated with almost identical film quality, gate dielectric/channel interface, and back-channel conditions. However, among various oxide TFTs using thickness-engineered InZnO thin films, only the 8 nm thick InZnO film exhibits excellent operational stability and electrical performance suitable for on/off switching and amplification functions, including high on/off current ratio (*I_ON/OFF_* = ~10^7^), superior mobility (*μ_FE_* = 2.1 cm^2^/V·s), and low sub-threshold swing value (*S.S.* = 0.289). In contrast, the ultrathin and thick films exhibit electrical performances that were either very resistive (high positive *V_Th_* and low on-current) or excessively conductive (high negative *V_Th_* and high off-current).

## Data Availability

Data are contained within the article and supplementary materials.

## Supplementary Materials

The following supporting information can be downloaded at: https://www.mdpi.com/article/10.3390/mi15020193/s1, Figure S1: Surface image and film thickness of thickness-controlled InZnO semiconductors acquired via AFM analysis; film thicknesses are (a) 4, (b) 8, (c) 12, and (d) 16 nm. Figure S2: XPS O 1s peak data and deconvoluted fitting plots of InZnO semiconductors with film thicknesses of (a) 4, (b) 8, (c) 12, and (d) 16 nm. Figure S3: Electrical characteristics of TFTs fabricated using thickness-controlled InZnO semiconductors: (a) 4 nm thick InZnO, (b) 8 nm thick InZnO, (c) 12 nm thick InZnO, and (d) 16 nm thick InZnO. Eleven devices were fabricated and measured for each channel thickness condition. Figure S4: Field-effect mobility (*μ_FE_*) of TFTs fabricated using thickness-controlled InZnO semiconductors: (a) 4 nm thick InZnO, (b) 8 nm thick InZnO, (c) 12 nm thick InZnO, and (d) 16 nm thick InZnO. Eleven devices were fabricated and measured for each channel thickness condition. Figure S5: The on/off current ratio (*I_ON/OFF_*) of TFTs fabricated using thickness-controlled InZnO semiconductors: (a) 4 nm thick InZnO, (b) 8 nm thick InZnO, (c) 12 nm thick InZnO, and (d) 16 nm thick InZnO. Eleven devices were fabricated and measured for each channel thickness condition. Figure S6: Threshold voltage (*V_Th_*) of TFTs fabricated using thickness-controlled InZnO semiconductors: (a) 4 nm thick InZnO, (b) 8 nm thick InZnO, (c) 12 nm thick InZnO, and (d) 16 nm thick InZnO. Eleven devices were fabricated and measured for each channel thickness condition. Figure S7: Electrical characteristics of TFTs fabricated using a single spin-coating of different concentrations of IZO solution (0.1 M to 0.4 M) obtain transistors with different InZnO thickness. Figure S8: Transfer curve shifts of TFTs fabricated using thickness-controlled InZnO semiconductors under NBS tests: (a) 4 nm thick InZnO, (b) 8 nm thick InZnO, (c) 12 nm thick InZnO, (d) 16 nm thick InZnO.
