# Supplementary material for: Solution Process-Based Thickness Engineering of InZnO Semiconductors for Oxide Thin-Film Transistors with High Performance and Stability
Source: Micromachines (Basel). 2024 Jan 27;15(2):193. doi: 10.3390/mi15020193 (PMC10890482; doi:10.3390/mi15020193)
Supplement: Supplementary file 1 [file micromachines-15-00193-s001.zip › micromachines-2826799-supplementary.pdf]

Supplementary Material

Solution Process-Based Thickness Engineering of InZnO Semiconductors for Oxide Thin-Film Transistors with High Performance and Stability

Xuan Zhang and Sung-Woon Cho *

Department of Advanced Components and Materials Engineering, Sunchon National University, Sunchon 57922, Republic of Korea
* Correspondence: swcho@scnu.ac.kr

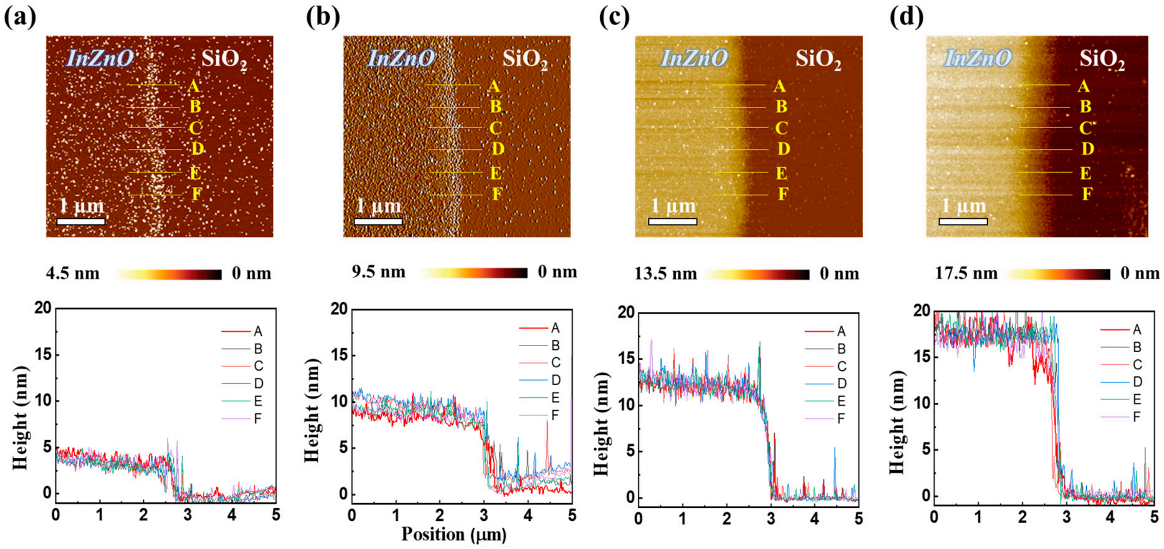

Figure S1. Surface image and film thickness of thickness-controlled InZnO semiconductors aquired via AFM analysis; film thicknesses are (**a**) 4, (**b**) 8, (**c**) 12, and (**d**) 16 nm.

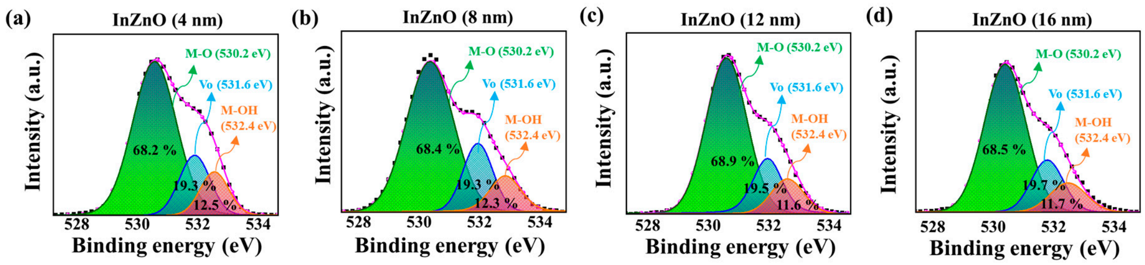

Figure S2. XPS O 1s peak data and deconvoluted fitting plots of InZnO semiconductors with film thicknesses of (**a**) 4, (**b**) 8, (**c**) 12, and (**d**) 16 nm.

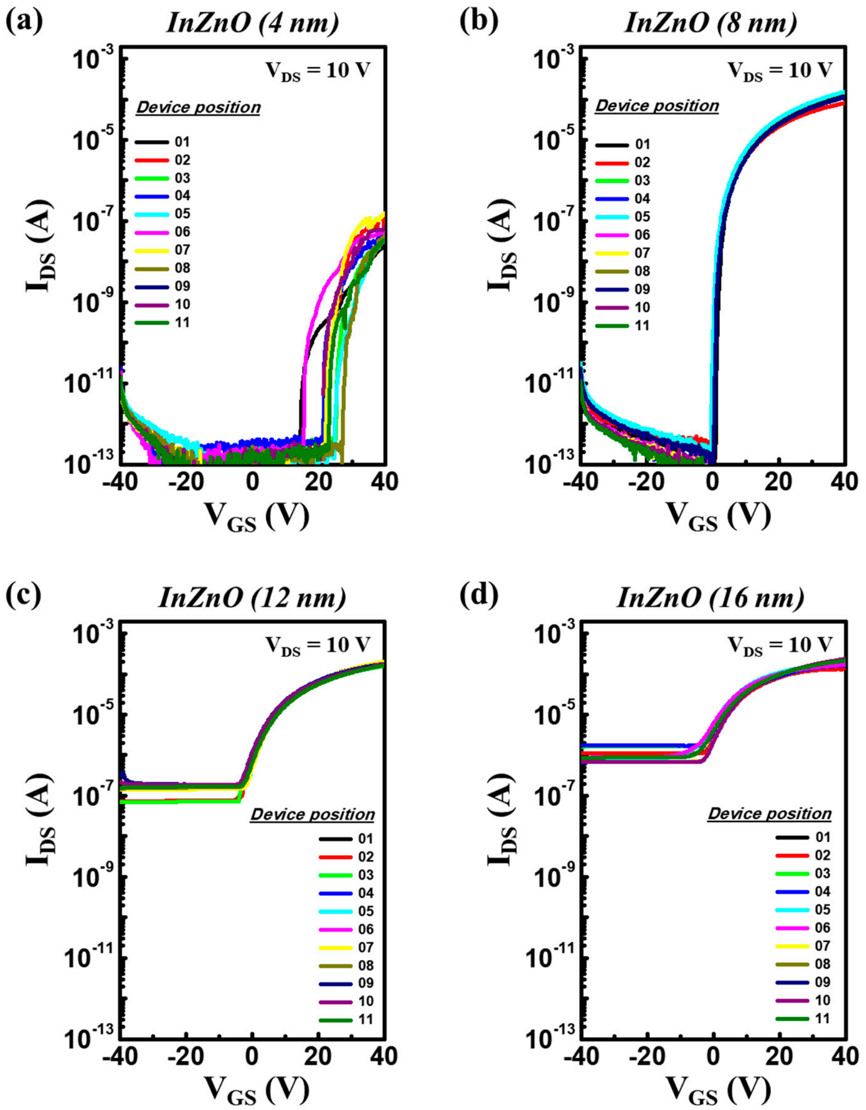

Figure S3. Electrical characteristics of TFTs fabricated using thickness-controlled InZnO semiconductors: (**a**) 4-nm-thick InZnO, (**b**) 8-nm-thick InZnO, (**c**) 12-nm-thick InZnO, and (**d**) 16-nm-thick InZnO. 11 devices were fabricated and measured for each channel thickness condition.

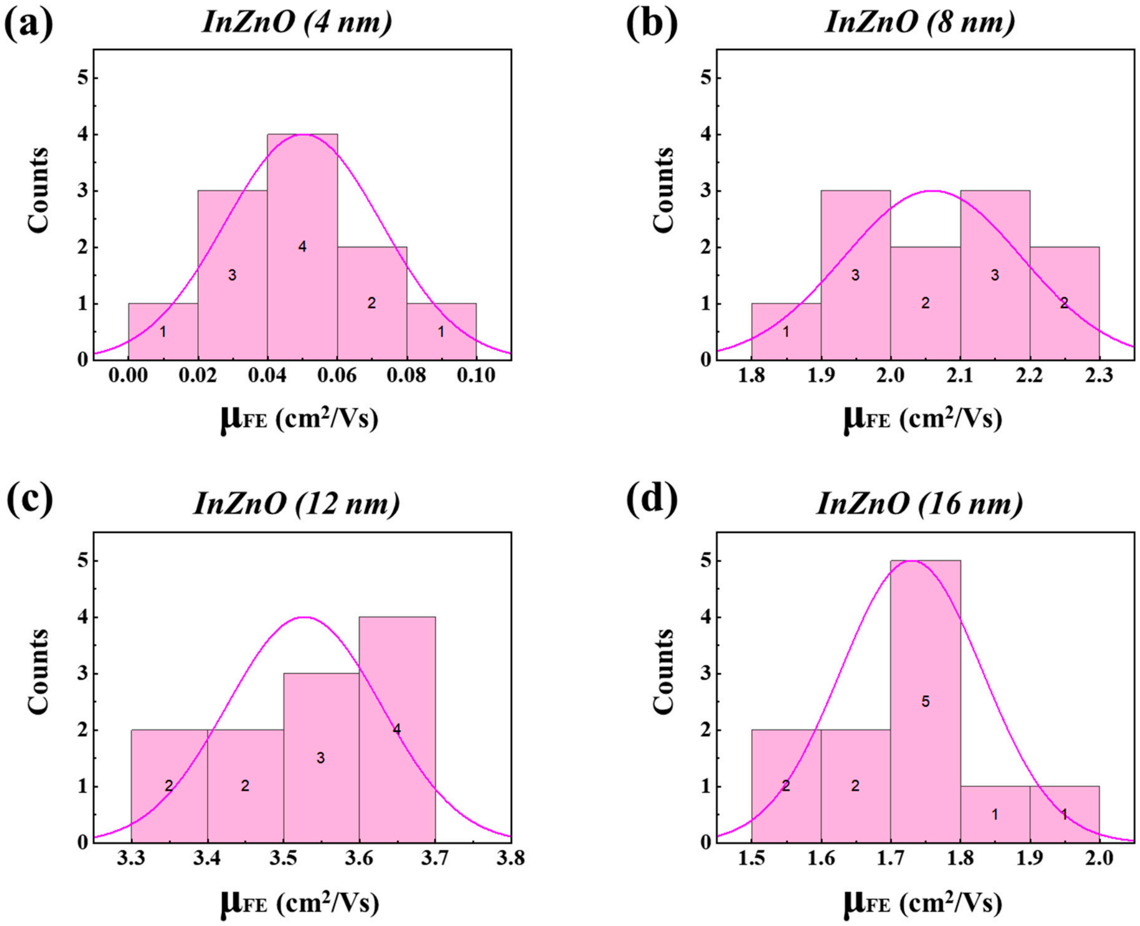

Figure S4. Field-effect mobility (μ_{FE}) of TFTs fabricated using thickness-controlled InZnO semiconductors: (**a**) 4-nm-thick InZnO, (**b**) 8-nm-thick InZnO, (**c**) 12-nm-thick InZnO, and (**d**) 16-nm-thick InZnO. 11 devices were fabricated and measured for each channel thickness condition.

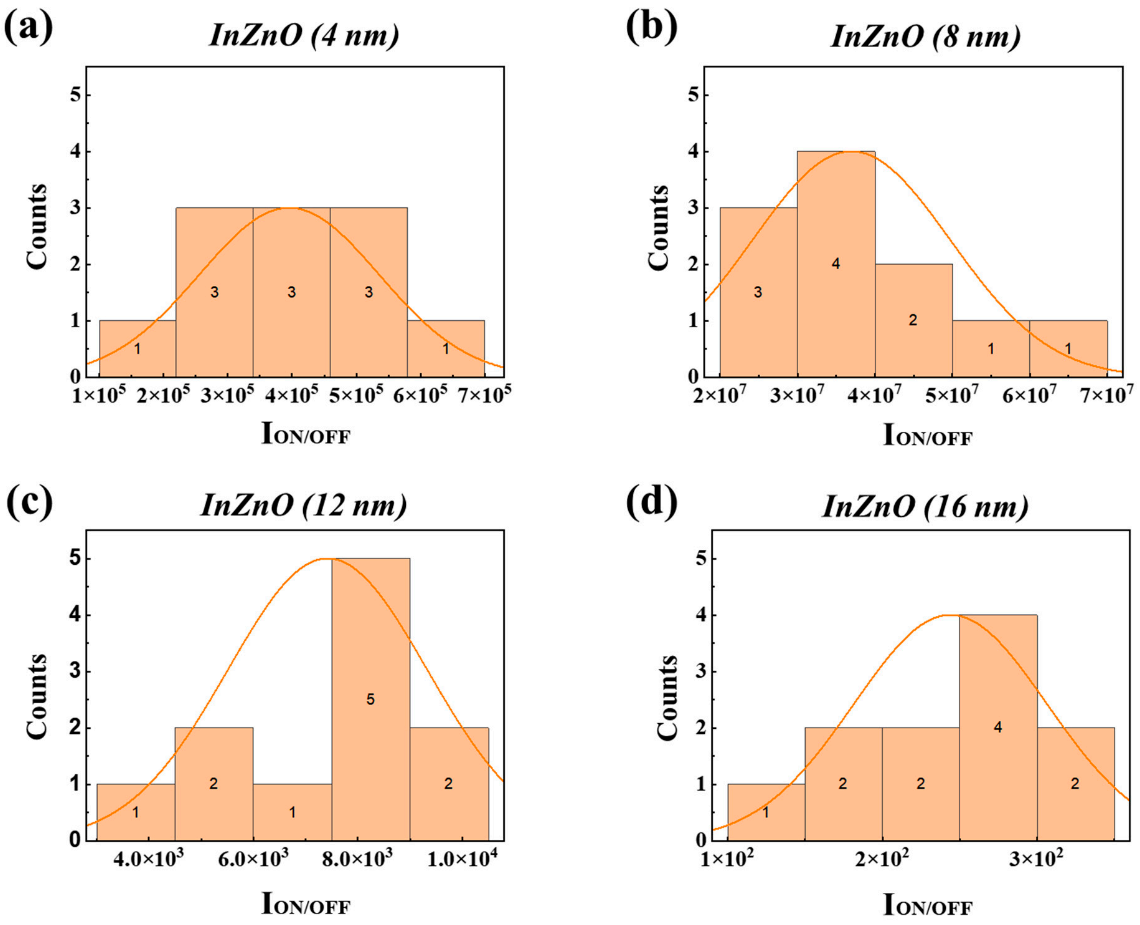

Figure S5. The on/off current ratio ($I_{ON/OFF}$) of TFTs fabricated using thickness-controlled InZnO semiconductors: (**a**) 4-nm-thick InZnO, (**b**) 8-nm-thick InZnO, (**c**) 12-nm-thick InZnO, and (**d**) 16-nm-thick InZnO. 11 devices were fabricated and measured for each channel thickness condition.

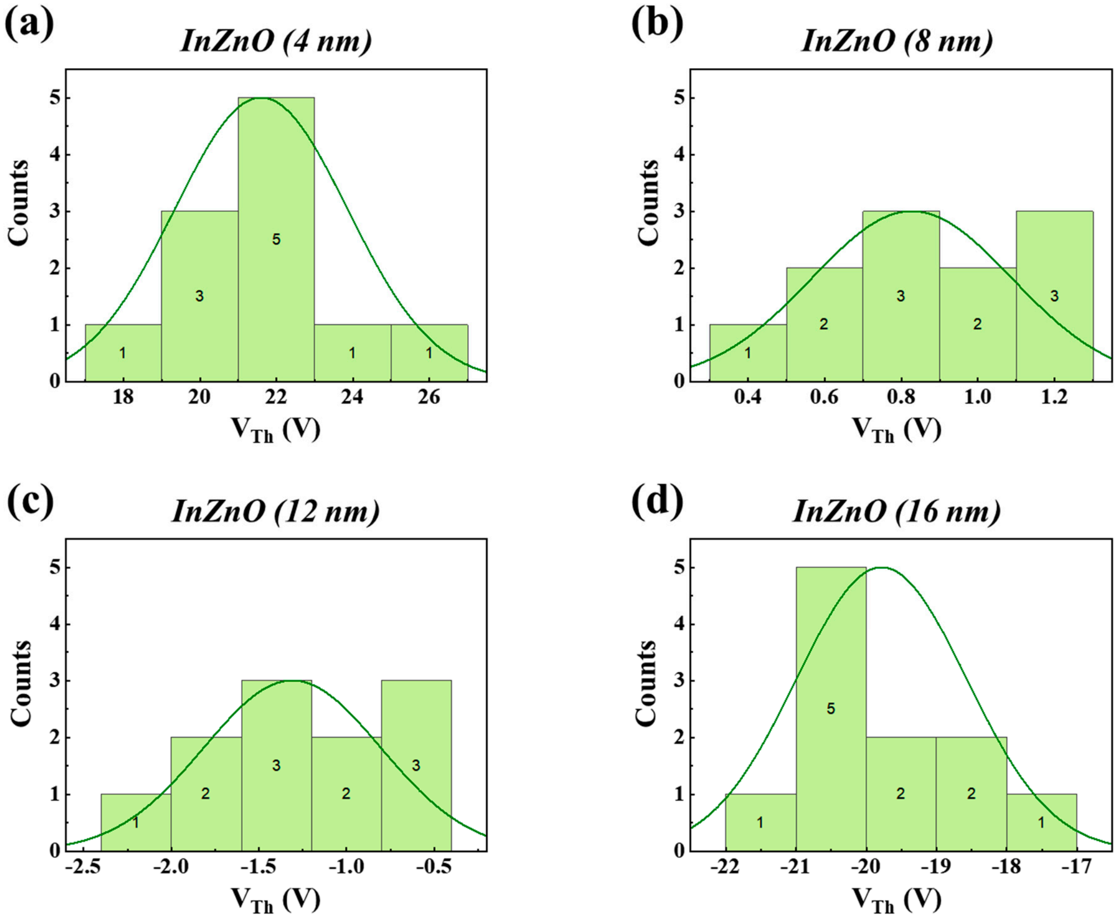

Figure S6. Threshold voltage (V_{Th}) of TFTs fabricated using thickness-controlled InZnO semiconductors: (**a**) 4-nm-thick InZnO, (**b**) 8-nm-thick InZnO, (**c**) 12-nm-thick InZnO, and (**d**) 16-nm-thick InZnO. 11 devices were fabricated and measured for each channel thickness condition.

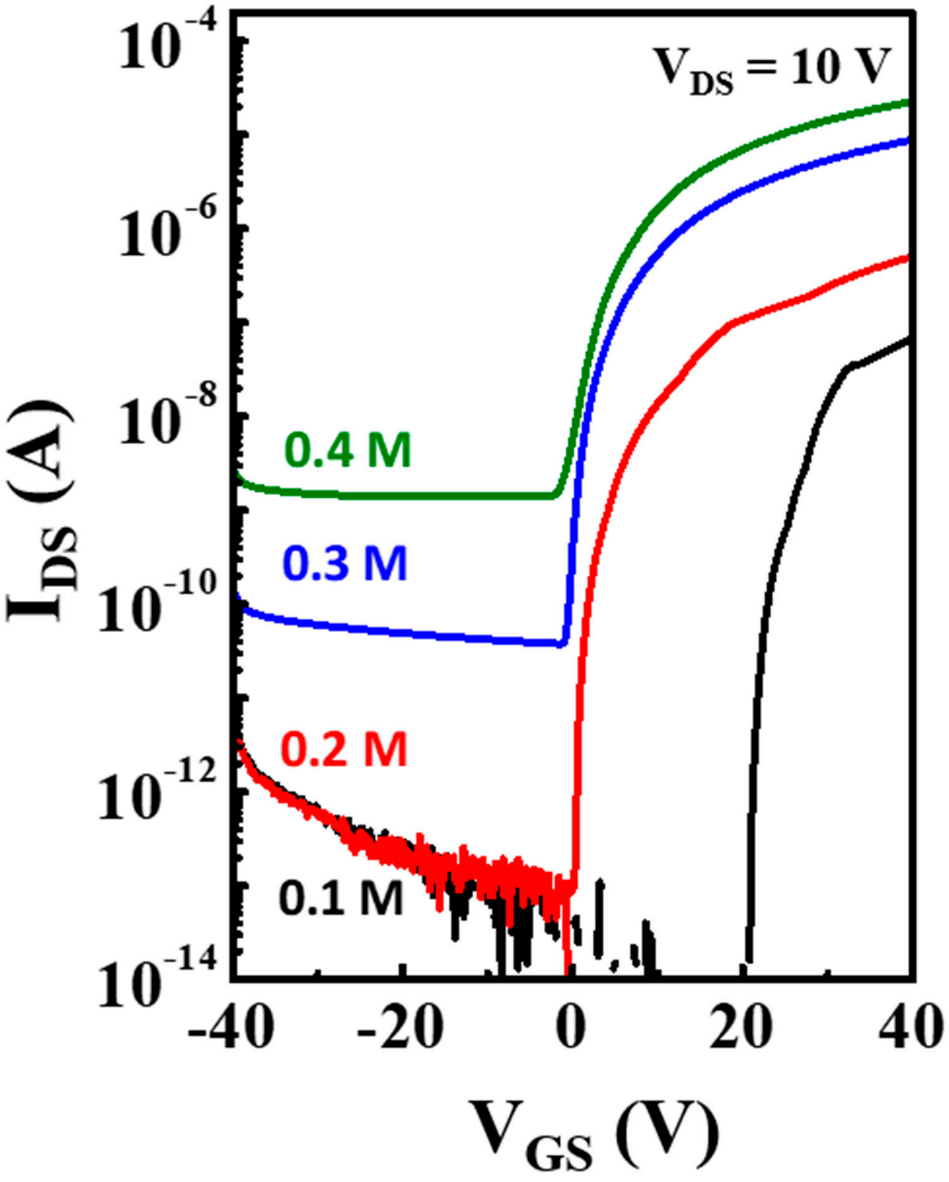

Figure S7. Electrical characteristics of TFTs fabricated using a single spin-coating of different concentrations of IZO solution (0.1 M to 0.4 M) obtain transistors with different InZnO thickness.

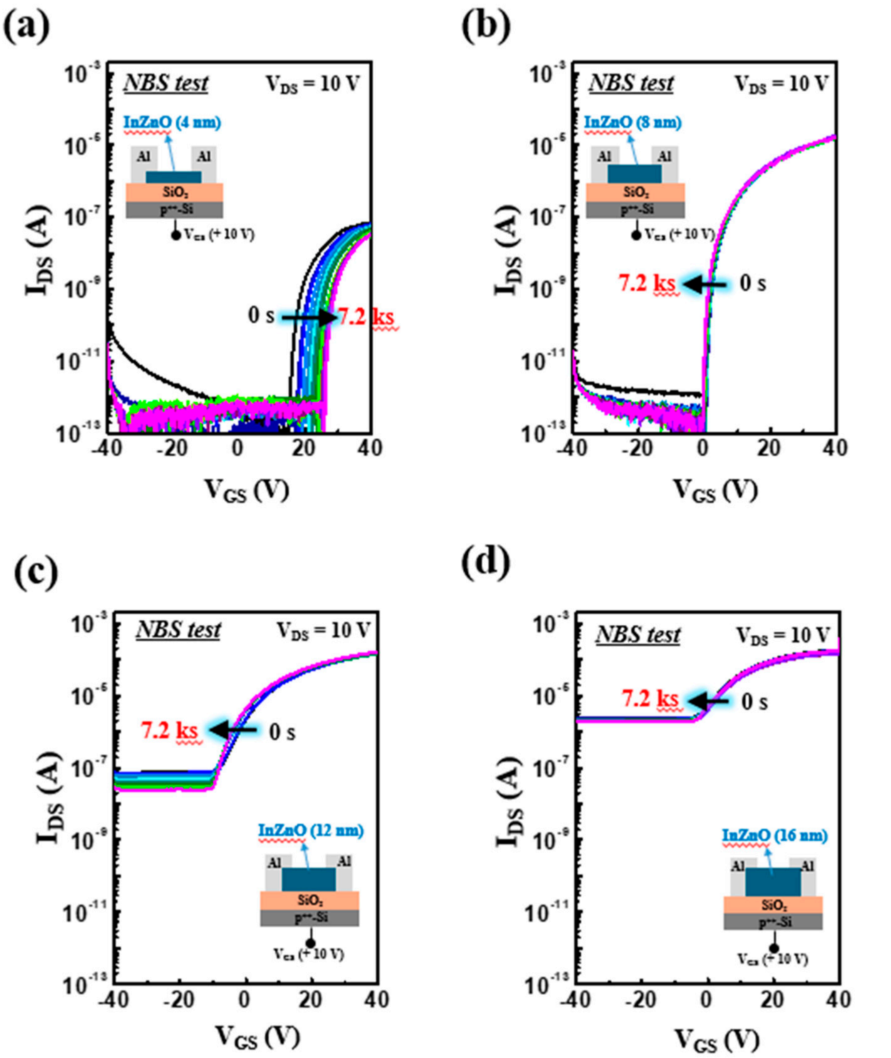

Figure S8. Transfer curve shifts of TFTs fabricated using thickness-controlled InZnO semiconductors under NBS tests: (**a**) 4-nm-thick InZnO, (**b**) 8-nm-thick InZnO, (**c**) 12-nm-thick InZnO, (d) 16-nm-thick InZnO.
